# COVID-19 Model with High- and Low-Risk Susceptible Population Incorporating the Effect of Vaccines

**DOI:** 10.3390/vaccines11010003

**Published:** 2022-12-20

**Authors:** Alhassan Ibrahim, Usa Wannasingha Humphries, Amir Khan, Saminu Iliyasu Bala, Isa Abdullahi Baba, Fathalla A. Rihan

**Affiliations:** 1 Department of Mathematics, Faculty of Science, King Mongkut’s University of Technology Thonburi (KMUTT), 126 Pracha Uthit Road, Bang Mod, Thrung Khru, Bangkok 10140, Thailand; 2 Department of Mathematical Sciences, Bayero University, Kano Kano 700006, Nigeria; 3 Department of Mathematics and Statistics, University of Swat, Khyber 01923, Pakistan; 4 Department of Mathematical Sciences, College of Science, UAE University, Al Ain 15551, United Arab Emirates; 5 Department of Mathematics, Faculty of Science, Helwan University, Cairo 11795, Egypt

**Keywords:** equilibrium solutions, stability analysis, COVID-19, global stability, sensitivity, vaccine

## Abstract

It is a known fact that there are a particular set of people who are at higher risk of getting COVID-19 infection. Typically, these high-risk individuals are recommended to take more preventive measures. The use of non-pharmaceutical interventions (NPIs) and the vaccine are playing a major role in the dynamics of the transmission of COVID-19. We propose a COVID-19 model with high-risk and low-risk susceptible individuals and their respective intervention strategies. We find two equilibrium solutions and we investigate the basic reproduction number. We also carry out the stability analysis of the equilibria. Further, this model is extended by considering the vaccination of some non-vaccinated individuals in the high-risk population. Sensitivity analyses and numerical simulations are carried out. From the results, we are able to obtain disease-free and endemic equilibrium solutions by solving the system of equations in the model and show their global stabilities using the Lyapunov function technique. The results obtained from the sensitivity analysis shows that reducing the hospitals’ imperfect efficacy can have a positive impact on the control of COVID-19. Finally, simulations of the extended model demonstrate that vaccination could adequately control or eliminate COVID-19.

## 1. Introduction

The COVID-19 pandemic is still among the most devastating infectious diseases in the world. It is caused by severe acute respiratory syndrome coronavirus 2 (SARS-CoV-2) and is transmitted within individuals through respiratory droplets created when someone that is infected coughs or sneezes or via direct contact with saliva, nasal discharge, and sputum of an infected individual [1,2]. The COVID-19 virus has been responsible for over 6 million deaths around the world by the end of March 2022, as a result, it has become the most significant global health disaster since the 1918 influenza pandemic [3,4].

The fight against COVID-19 made significant strides in early 2022 when the reported daily number of cases plunged, and hospitalizations were down by almost 27.9%. These changes are attributed to the wide implementation of COVID-19 control strategies on a large scale, which includes keeping in touch with the authorities, getting vaccinated, properly wearing a recommended facemask, avoiding crowded areas, or maintaining distance between the self and others, yet an infection is still detected [5,6]. Therefore, it is reasonable to speculate that transmission cannot be ruled out completely, and given the potential importance of such transmission, urgent research on this subject is imperative [7,8].

Although COVID-19 vaccines are readily available, there is no antiviral treatment that has been recommended at this time for any of the COVID-19 variants [9]. Several clinical trials are being conducted to evaluate the effectiveness of potential treatments. Despite this, there are treatments available that can help relieve symptoms and help shorten the length of the illness as well [10,11]. There are a few treatments that are aimed at relieving symptoms, as well as supporting the respiratory system. Hospitalization may be necessary for some people, especially if they have an underlying medical condition that requires treatment [12].

There are more than 30 COVID-19 vaccines approved by National regulatory authorities around the world in which most of them are proven to be effective [13]. Additionally, more than half of the world’s population received at least one dose of the vaccine [14]. Many people refused to take the optimal dosage of the vaccine due to various reasons, including due to fake information related to its safety and long-term effects. Whatever the reason, there are significant consequences when individuals opt out of vaccines [15]. Outbreaks of COVID-19 are more likely to occur in communities where vaccination rates are low. This not only puts unvaccinated individuals at risk but also increases the chances that these diseases will spread to other people, including low-risk individuals.

The COVID-19 vaccines are the subject of extensive debate on the ideal dosage that would offer the greatest degree of protection. It has been claimed by several researchers that taking the booster dose is crucial for managing the condition and preventing major illness or even death induced by this disease. As an interim measure, the most effective way to curb the spread of this disease is through vaccination on a regular basis and in accordance with the recommended schedule [16].

There has been numerous studies conducted to better understand how the virus spreads from one person to another. The use of mathematical models to predict disease transmission is beneficial for analysis and prediction, especially when tested against accessible disease data. The initial models generally consist of deterministic ordinary differential equations of the SEIR form which are rigorously analyzed by different authors taking different stages into account. See, for instance, Swati et al. in [17] analyze the COVID-19 model considering hospitalized individuals, and home-quarantined, or home-treated individuals. According to Iboi [18], a mathematical model was developed for evaluating the influence of the NPIs on the transmission pattern of the COVID-19 pandemic, as well as investigating the impact of the early removal of social distancing and community lockdown measures. Their work was extended by Riyapan et al. in [19], in which he added a compartment.

Tylicki et al. in [20] shows that the susceptible class is divided into two: the high risk and the low risk. They indicated that the elderly, males, hypertensive, and patients with comorbidities, constitute the most well-characterized high-risk group for severe manifestations of COVID-19. None of the research mentioned above considered this division in detail mathematically.

Understanding the dynamics of the interactions between those at low risk and those at high risk is crucial to the control and prevention of COVID-19. This requires some mathematical models, which have the potential to enhance our understanding of the parameters associated with the increase in infection rates. Hence, our aim in this paper is to examine the dynamics of COVID-19 transmission in two susceptible populations setting (low- and high-risk individuals) through mathematical modeling, where we also use the non-linear expression of the incidence rate. This incidence rate expression is proved to be more realistic than the corresponding bilinear incidence rate [21].

Here, we use the COVID-19 features to develop a model and utilized some data that are either real, estimated, or from the literature. In order to identify the most sensitive parameter associated with the model basic reproduction number $R_{0}$, a sensitivity analysis was conducted.

We organize this paper as follows: in Section 1, we give the introduction; Section 2 is the model formulation; in Section 3, we analyse the model qualitatively, which includes computing the reproduction number using a technique called the next generation matrix, calculating disease-free equilibrium points, identifying endemic equilibrium points, analysing the stability of these equilibrium points on a local and global scale. In Section 4, we perform sensitivity analysis and numerical simulations of the model; in Section 5, we extend the model by considering movement from the high-risk to low-risk susceptible population which is caused due to vaccination of some unvaccinated individuals in the high-risk population. We provide discussion and conclusions regarding our results in Section 6.

## 2. Model Formulation

In this part, we modify an existing COVID-19 model proposed by Pal Bajiya et al. in [22]. The modification procedure is presented below. We define N(t) as the total human population at time *t* divided into six sub-populations: high-risk susceptible $S_{1}$, low-risk susceptible $S_{2}$, exposed *E*, infected *I*, hospitalized *H*, and recovered *R* individuals:

(1) $$N\left(t\right)=S_{1}\left(t\right)+S_{2}\left(t\right)+E\left(t\right)+I\left(t\right)+H\left(t\right)+R\left(t\right).$$

Our model also accounts for certain demographic impacts by assuming that all sub-populations have a natural death rate (μ>0) and a net inflow of humans to the two susceptible population at a rate Λ. Next, we define the incident fraction as follows:

(2) $$\eta =\frac{\beta (I+\epsilon H)}{N},$$

where β is the effective contact rate, and ϵ is a modification factor that measures hospital inefficacy.

The high-risk susceptible population $S_{1}\left(t\right)$ are those that are unvaccinated, human with underlying medical conditions demonstrated to have a higher risk of death due to COVID-19 [23], as well as the elderly [24]. Undocumented migrants associated with limited access to healthcare because of legal, administrative, social barriers, etc., are also part of this population. The high-risk susceptible population $S_{1}\left(t\right)$ is reduced by the natural death rate μ and the force of infection η. The parameter $\sigma_{1}$ incorporates the impact of the non-pharmaceutical intervention (wearing masks, physical or social distancing, and washing hands regularly) by individuals in $S_{1}$ on the number of contacts,

$$\frac{dS_{1}}{dt}=\left(1-\rho \right)\Lambda -\left(\left(1-\sigma_{1}\right)\eta +\mu \right)S_{1}.$$

Similarly, the low-risk susceptible population $S_{2}\left(t\right)$ includes those that are not in $S_{1}\left(t\right)$, and are recruited either by birth, or screened of COVID-19 by the authorities at the point of entry through any of the borders. $S_{2}\left(t\right)$ also reduced by natural death rate μ and force of infection η, following effective contacts with an infected individual. Here, $\sigma_{2}$ represents the same interventions with $S_{1}$. High-risk individuals must take additional precautions in addition to the above-mentioned actions to reduce transmission, this implies $0\leq \sigma_{2}<\sigma_{1}$,

$$\frac{dS_{2}}{dt}=\rho \Lambda -\left(\left(1-\sigma_{2}\right)\eta +\mu \right)S_{2}.$$

Exposed individuals E(t) stands for the number of people who were exposed to the virus. They are generated by the population of susceptible (high or low risk) humans that become exposed or contact with the infected persons, and decreases as a result of infection at a rate α and natural death at the rate μ,

$$\frac{dE}{dt}=\eta \left(\left(1-\sigma_{1}\right)S_{1}+\left(1-\sigma_{2}\right)S_{2}\right)-\left(\alpha +\mu \right)E.$$

Infected individuals I(t) stands for the number of people who are infected but have not been detected. It is reduced by either hospitalization at the rate of $\nu_{2}$, recovery without being hospitalized at a rate $\nu_{1}$, the natural death μ, and COVID-19 induced death at a rate δ,

$$\frac{dI}{dt}=\alpha E-\left(\nu_{1}+\nu_{2}+\mu +\delta \right)I.$$

Hospitalized individuals H(t) are those who have been diagnosed with COVID-19 and have been isolated by the authorities. They are reduced due to recovery at the rate τ, the natural death μ, and COVID-19 induced death at a rate δ,

$$\frac{dH}{dt}=\nu_{2}I-\left(\tau +\mu +\delta \right)H.$$

Recovered individuals R(t) represents the number of recovered, undiagnosed individuals, who are not being officially identified and those that recovered due to the impact of isolation. The only decline in this population is due to the natural death rate μ,

$$\frac{dR}{dt}=\nu_{1}I+\tau H-\mu R.$$

**Figure 1:**
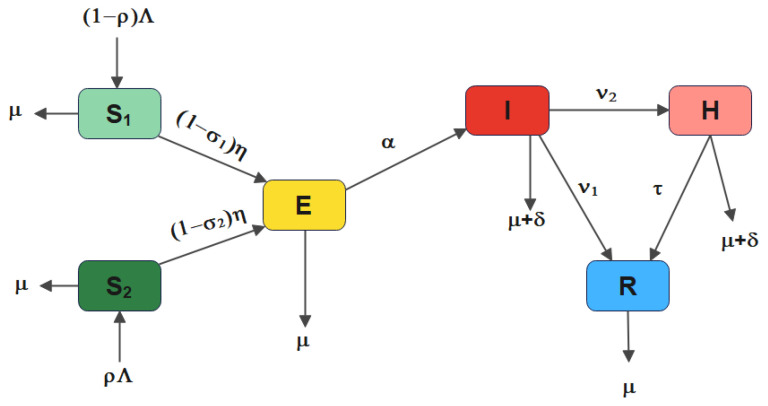
Schematic diagram of the COVID-19 model.

Table 1 contains the model’s state variables, whereas Table 2 lists the model parameters and their descriptions. Based on the assumptions stated above, Figure 1, below, describes the flow transmission of the infection from one compartment to another. The interactions is represented by the system of non-linear ordinary differential equations below:

(3) $$\begin{array}{cc} \frac{dS_{1}}{dt} & =\left(1-\rho \right)\Lambda -\left(\left(1-\sigma_{1}\right)\eta +\mu \right)S_{1},\\ \frac{dS_{2}}{dt} & =\rho \Lambda -\left(\left(1-\sigma_{2}\right)\eta +\mu \right)S_{2},\\ \frac{dE}{dt} & =\eta \left(\left(1-\sigma_{1}\right)S_{1}+\left(1-\sigma_{2}\right)S_{2}\right)-\left(\alpha +\mu \right)E,\\ \frac{dI}{dt} & =\alpha E-(\nu_{1}+\nu_{2}+\mu +\delta )I,\\ \frac{dH}{dt} & =\nu_{2}I-\left(\tau +\mu +\delta \right)H,\\ \frac{dR}{dt} & =\nu_{1}I+\tau H-\mu R. \end{array}$$

The basic dynamic characteristics of the model will now be discussed. Since the model tracks populations of humans. In the same way as [25], the following theorem shows that all the state variables are non-negative for all time t>0.

**Theorem** **1.** *Consider the model (3) with initial conditions $S_{1}\left(0\right)>0,S_{2}\left(0\right)\geq 0$, E(0)≥0, I(0)≥0,H(0)≥0, and R(0)≥0 then the solution is positive in $R_{+}^{6}$.*

**Proof.**  Using the first equation of the model (3) given by

$$\frac{dS_{1}}{dt}=\left(1-\rho \right)\Lambda -\left(\left(1-\sigma_{1}\right)\eta +\mu \right)S_{1},$$

we have

$$\frac{dS_{1}}{dt}\geq -\left(\left(1-\sigma_{1}\right)\eta +\mu \right)S_{1}.$$

Solving for $S_{1}$

$$S_{1}\left(t\right)\geq S_{1}\left(0\right)e^{-\int (\left(1-\sigma_{1}\right)\eta +\mu )dt},$$

which implies that

$$S_{1}\left(t\right)\geq 0,\;\mathrm{since}\;(\left(1-\sigma_{1}\right)\eta +\mu )>0$$.

Similarly, $S_{2}\left(t\right),E\left(t\right),I\left(t\right),H\left(t\right),R\left(t\right)$ can be demonstrated to be positive. Therefore, the solution of the model (3) is a positive quantity in $R_{+}^{6}$ for all t≥0. □

**Theorem** **2.** *The model system’s (3) solution is bounded in*

$$D=\{\left(S_{1},S_{2},E,I,H,R\right)\in R_{+}^{6}|0:N\leq \frac{\Lambda }{\mu }\}.$$

**Proof.**  By combining all equations in (3) we obtain

$$\frac{dN}{dt}=\Lambda -\mu N-\delta \left(I+H\right)\leq \Lambda -\mu N,$$

$$\frac{dN}{dt}+\mu N\leq \Lambda ,$$

therefore

$$N\left(t\right)\leq N\left(0\right)e^{-\mu t}+\frac{\Lambda }{\mu }\left(1-e^{-\mu t}\right),$$

where N(0) is the initial values, i.e., N(t)=N(0)att=0.

Following from [26], it can be observed that $N\left(t\right)⟶\frac{\Lambda }{\mu }$ as t⟶∞. Hence, N(t) is bounded as $0\leq N\left(t\right)\leq \frac{\Lambda }{\mu }$. So, all solutions in $R_{+}^{6}$ of the model (3) eventually enter in D. □

The proposed model is well presented epidemiologically and mathematically from above in the region D. As a result, analysing the qualitative dynamics of model in D is adequate.

## 3. Model Analysis

The model system (3) admits two equilibria: disease free equilibrium (DFE) and endemic equilibrium (EE).

### 3.1. Disease Free Equilibrium

If there is no disease, the DFE of the model system (3) is calculated as follows:

Setting E=0,I=0,H=0,R=0 and denoted as

$$DFE=\left(S_{1}^{0},S_{2}^{0},E^{0},I^{0},H^{0},R^{0}\right)=\left(\frac{(1-\rho )\Lambda }{\mu },\frac{\rho \Lambda }{\mu },0,0,0,0\right).$$

We computed the basic reproduction number in a similar manner as in [27]. Considering the infected block x={E,I,H} we obtain the matrix FandV as follows:

(4) $$F=\left(\begin{array}{ccc} 0 & \beta (\left(1-\sigma_{1}\right)\left(1-\rho \right)+\left(1-\sigma_{2}\right)\rho ) & \beta \epsilon (\left(1-\sigma_{1}\right)\left(1-\rho \right)+\left(1-\sigma_{2}\right)\rho )\\ 0 & 0 & 0\\ 0 & 0 & 0 \end{array}\right),$$

(5) $$V=\left(\begin{array}{ccc} \alpha +\mu & 0 & 0\\ -\alpha & \nu_{1}+\nu_{2}+\mu +\delta & 0\\ 0 & -\nu_{2} & \tau +\mu +\delta \end{array}\right).$$

Therefore, the basic reproduction $R_{0}$ is as follows

(6) $$R_{0}=\frac{\alpha \beta \left(\epsilon \nu_{2}+\tau +\mu +\delta \right)\left(\left(\sigma_{1}-\sigma_{2}\right)\rho +\left(1-\sigma_{1}\right)\right)}{\left(\nu_{1}+\nu_{2}+\mu +\delta \right)\left(\alpha +\mu \right)\left(\tau +\mu +\delta \right)}.$$

The basic reproduction number $R_{0}$ defined as the number of new infections in a population induced by a single infected person within a given period of time. If the $R_{0}<1$, the DFE is said to be locally stable, otherwise is said to be unstable [17].

**Theorem** **3.** *The disease free equilibrium point DFE of the model (3) is said to be locally asymptotically stable (LAS) if $R_{0}<1$ and unstable if $R_{0}>1$.*

**Proof.**  From the Jacobian matrix evaluated at the DFE defined as

(7) $$J_{DFE}=\left[\begin{array}{cccccc} -\mu & 0 & 0 & -\beta (\sigma 1-1)(\rho -1) & -\beta \epsilon \left(\sigma_{1}-1\right)\left(\rho -1\right) & 0\\ 0 & -\mu & 0 & \beta \rho (\sigma_{2}-1) & \beta \epsilon \rho (\sigma_{2}-1) & 0\\ 0 & 0 & -(\alpha +\mu ) & \beta (\left(\sigma_{1}-\sigma_{2}\right)\rho +\left(1-\sigma_{1}\right)) & \beta \epsilon (\left(\sigma_{1}-\sigma_{2}\right)\rho +\left(1-\sigma_{1}\right)) & 0\\ 0 & 0 & \alpha & -(\mu +\delta +\nu_{1}+\nu_{2}) & 0 & 0\\ 0 & 0 & 0 & \nu_{2} & -(\mu +\tau +\delta ) & 0\\ 0 & 0 & 0 & \nu_{1} & \tau & -\mu \end{array}\right].$$

λ=−μ is a negative root of multiplicity 3 $\left(\lambda_{1}=\lambda_{2}=\lambda_{6}=-\mu \right)$. A reduced Jacobian matrix defined as $J_{r_{DFE}}$ below has three (3) eigenvalues, which correspond to the remaining eigenvalues of $J_{DFE}$.

(8) $$J_{r_{DFE}}=\left[\begin{array}{ccc} -(\alpha +\mu ) & \beta (\left(\sigma_{1}-\sigma_{2}\right)\rho +\left(1-\sigma_{1}\right)) & \beta \epsilon (\left(\sigma_{1}-\sigma_{2}\right)\rho +\left(1-\sigma_{1}\right))\\ \alpha & -(\mu +\delta +\nu_{1}+\nu_{2}) & 0\\ 0 & \nu_{2} & -(\mu +\tau +\delta ) \end{array}\right].$$

Define $k_{1}=\alpha +\mu$, $k_{2}=\left(\sigma_{1}-\sigma_{2}\right)\rho +\left(1-\sigma_{1}\right)$, $k_{3}=\nu_{1}+\nu_{2}+\mu +\delta$, and $k_{4}=\mu +\tau +\delta$. The characteristic polynomial of the matrix $Jr_{DFE}$ is determined as

(9) $$\lambda^{3}+c_{1}\lambda^{2}+c_{2}\lambda +c_{3}=0.$$

where

$c_{1}=k_{4}+k_{3}+k_{1},$
$c_{2}=k_{1}k_{3}+k_{1}k_{4}+k_{3}k_{4}-\alpha \beta k_{2},$
$c_{3}=k_{1}k_{3}k_{4}-\alpha \beta k_{2}k_{4}-\alpha \beta \epsilon \nu_{2}k_{2}=k_{1}k_{3}k_{4}-\alpha \beta k_{2}\left(k_{4}+\epsilon \nu_{2}\right)$
$=k_{1}k_{3}k_{4}\left(1-\frac{\alpha \beta k_{2}\left(k_{4}+\epsilon \nu_{2}\right)}{k_{1}k_{3}k_{4}}\right)$
$=k_{1}k_{3}k_{4}\left(1-R_{0}\right).$

According to Routh–Hurwitz criterion, the sufficient and necessary condition for stability is

(10) $$c_{1}>0,c_{3}>0,c_{1}c_{2}-c_{3}>0.$$

Because all of the model parameters are positive, the first inequality in (10) is automatically satisfied, the second inequality is satisfied when $R_{0}<1$. As a result $c_{1}c_{2}-c_{3}>0$ as long as $c_{3}>0$ holds. Hence, the disease free equilibrium DFE of the model (3) is locally asymptotically stable when $R_{0}<1$ and unstable whenever $R_{0}>1$. □

For global stability of the DFE, we have the following theorem

**Theorem** **4.** *The disease-free equilibrium (DFE) of the model (3) is globally asymptotically stable (GAS) if $R_{0}<1.$*

**Proof.**  Considering the following Voltera-type Lyapunov function

(11) $$L_{0}=\left(S_{1}-S_{1_{0}}\ln\left(S_{1}\right)\right)+\left(S_{2}-S_{2_{0}}\ln\left(S_{2}\right)\right)+f_{1}E+f_{2}I+f_{3}H$$

where $f_{1},f_{2},\;\mathrm{and}\;f_{3}>0$ are the Lyapunov coefficient. The corresponding derivative of $L_{0}$$\left(\frac{dL_{0}}{dt}\right)$ is given by

(12) $$\dot{L_{0}}=-\frac{\mu {\left(S_{1}-S_{1_{0}}\right)}^{2}}{S_{1}}-\frac{\mu {\left(S_{2}-S_{2_{0}}\right)}^{2}}{S_{2}}+f_{1}\dot{E}+f_{2}\dot{I}+f_{3}\dot{H}.$$

(13) $$\begin{array}{cc} \dot{L_{0}} & =-\frac{\mu {\left(S_{1}-S_{1_{0}}\right)}^{2}}{S_{1}}-\frac{\mu {\left(S_{2}-S_{2_{0}}\right)}^{2}}{S_{2}}+f_{1}\left(\eta \left(1-\sigma_{1}\right)S_{1}+\eta \left(1-\sigma_{2}\right)S_{2}-k_{1}E\right)\\ \; & +f_{2}\left(\alpha E-k_{3}I\right)+f_{3}\left(\nu_{2}I-k_{4}H\right). \end{array}$$

At the DFE, we linearize the (13). We note that near the DFE,

$$S_{1}\leq \frac{(1-\rho )\Lambda }{\mu },S_{2}\leq \frac{\rho \Lambda }{\mu }\;\mathrm{and}\;\mathrm{therefore}\;\frac{S_{1}}{N}\leq \left(1-\rho \right)\;\mathrm{and}\;\frac{S_{2}}{N}\leq \rho$$.

Using this relation, we have

(14) $$\begin{array}{cc} \dot{L_{0}} & \leq -\frac{\mu {\left(S_{1}-S_{1_{0}}\right)}^{2}}{S_{1}}-\frac{\mu {\left(S_{2}-S_{2_{0}}\right)}^{2}}{S_{2}}+f_{1}(\beta \left(I+\epsilon H\right)\left(1-\rho \right)\left(1-\sigma_{1}\right)+\beta \left(I+\epsilon H\rho \left(1-\sigma_{2}\right)\right)\\ \; & -f_{1}k_{1}E+f_{2}\left(\alpha E-k_{3}I\right)+f_{3}\left(\nu_{2}I-k_{4}H\right). \end{array}$$

(15) $$\begin{array}{cc} \dot{L_{0}} & \leq -\frac{\mu {\left(S_{1}-S_{1_{0}}\right)}^{2}}{S_{1}}-\frac{\mu {\left(S_{2}-S_{2_{0}}\right)}^{2}}{S_{2}}+f_{1}\left(\beta \left(I+\epsilon H\right)k_{2}-k_{1}E\right)+f_{2}\left(\alpha E-k_{3}I\right)\\ \; & +f_{3}\left(\nu_{2}I-k_{4}H\right). \end{array}$$

We choose

$$f_{1}=\alpha ,f_{2}=k_{1},\;\mathrm{and}\;f_{3}=\frac{\alpha \beta \epsilon k_{2}}{k_{4}}$$

(15) becomes,

(16) $$\begin{array}{cc} \dot{L_{0}} & \leq -\frac{\mu {\left(S_{1}-S_{1_{0}}\right)}^{2}}{S_{1}}-\frac{\mu {\left(S_{2}-S_{2_{0}}\right)}^{2}}{S_{2}}+\alpha \left(\beta \left(I+\epsilon H\right)k_{2}-k_{1}E\right)+k_{1}\left(\alpha E-k_{3}I\right)\\ \; & +\frac{\epsilon \alpha }{k_{4}}\left(\nu_{2}I-k_{4}H\right). \end{array}$$

(17) $$=-\frac{\mu {\left(S_{1}-S_{1_{0}}\right)}^{2}}{S_{1}}-\frac{\mu {\left(S_{2}-S_{2_{0}}\right)}^{2}}{S_{2}}+Ik_{1}k_{3}\left[\frac{\alpha \beta (\epsilon \nu_{2}+k_{4})}{k_{1}k_{3}k_{4}}-1\right].$$

(18) $$=-\frac{\mu {\left(S_{1}-S_{1_{0}}\right)}^{2}}{S_{1}}-\frac{\mu {\left(S_{2}-S_{2_{0}}\right)}^{2}}{S_{2}}+Ik_{1}k_{3}\left[R_{0}-1\right].$$

Clearly, $\frac{dL_{0}}{dt}\leq 0$ whenever $R_{0}\leq 0$. As a result, and according to the Lasalle invariance principle [28], the DFE is said to be globally asymptotically stable. □

### 3.2. Endemic Equilibrium

Finding the endemic equilibrium solutions $S_{1}^{*},S_{2}^{*},E^{*},I^{*},H^{*},$ and $R^{*}$ defined as EE helps to further analyze the model. In order to obtain this endemic equilibrium, we solve (3) substituting $k_{1},k_{2},k_{3},\;\mathrm{and}\;k_{4}$ as defined in Section 3.1 simultaneously in terms η (i.e., force of infection) and obtained

(19) $$\begin{array}{cc} S_{1}^{*} & =\frac{\Lambda (1-\rho )}{\left(1-\sigma_{1}\right)\eta^{*}+\mu },\\ S_{2}^{*} & =\frac{\rho \Lambda }{\left(1-\sigma_{2}\right)\eta^{*}+\mu },\\ E^{*} & =\frac{\eta^{*}\Lambda \left(\left(1-\sigma_{2}\right)\left(1-\sigma_{1}\right)\eta^{*}+k_{2}\mu \right)}{k_{1}\left(\left(1-\sigma_{1}\right)\eta^{*}+\mu \right)\left(\left(1-\sigma_{2}\right)\eta^{*}+\mu \right)},\\ I^{*} & =\frac{\alpha \eta^{*}\Lambda \left(\left(1-\sigma_{2}\right)\left(1-\sigma_{1}\right)\eta^{*}+k_{2}\mu \right)}{k_{1}k_{3}\left(\left(1-\sigma_{1}\right)\eta^{*}+\mu \right)\left(\left(1-\sigma_{2}\right)\eta^{*}+\mu \right)},\\ H^{*} & =\frac{\alpha \nu_{2}\eta^{*}\Lambda \left(\left(1-\sigma_{2}\right)\left(1-\sigma_{1}\right)\eta^{*}+k_{2}\mu \right)}{k_{1}k_{3}k_{4}\left(\left(1-\sigma_{1}\right)\eta^{*}+\mu \right)\left(\left(1-\sigma_{2}\right)\eta^{*}+\mu \right)},\\ R^{*} & =\frac{\alpha \eta^{*}\Lambda \left(k_{4}\nu_{1}+\tau \nu_{2}\right)\left(\left(1-\sigma_{2}\right)\left(1-\sigma_{1}\right)\eta^{*}+k_{2}\mu \right)}{\mu k_{1}k_{3}k_{4}\left(\left(1-\sigma_{1}\right)\eta^{*}+\mu \right)\left(\left(1-\sigma_{2}\right)\eta^{*}+\mu \right)}. \end{array}$$

where

(20) $$\eta^{*}=\frac{\beta (I^{*}+\epsilon H^{*})}{N^{*}},$$

and $N^{*}=S_{1}^{*}+S_{2}^{*}+E^{*}+I^{*}+H^{*}+R^{*}$

From Equation (20) we obtained $\eta^{*}=0$ as one of the solutions (which corresponds to DFE) and the following equation which is quadratic:

(21) $$a\eta^{*2}+b\eta^{*}+c=0.$$

where

$a=k_{5}k_{6}\left(\alpha \mu k_{4}+\alpha \mu \nu_{2}+\alpha \tau \nu_{2}+\alpha k_{4}\nu_{1}+\mu k_{3}k_{4}\right),$
$b=\mu \rho k_{1}k_{3}k_{4}k_{5}+\alpha \mu^{2}k_{2}k_{4}+\alpha \mu^{2}k_{2}\nu_{2}+\alpha \mu \tau k_{2}\nu_{2}+\alpha \mu k_{2}k_{4}\nu_{1}+\mu^{2}k_{2}k_{3}k_{4}+\mu k_{1}k_{3}k_{4}k_{6}-$$\beta \alpha \epsilon \mu k_{5}k_{6}\nu_{2}-\beta \alpha \mu k_{4}k_{5}k_{6}-\mu \rho k_{1}k_{3}k_{4}k_{6},$
$c=\mu^{2}k_{1}k_{3}k_{4}-\beta \alpha \epsilon \mu^{2}k_{2}\nu_{2}-\beta \alpha \mu^{2}k_{2}k_{4}=\mu^{2}k_{1}k_{3}k_{4}\left(1-R_{0}\right),$

where $k_{5}=1-\sigma_{1}$ and $k_{6}=1-\sigma_{2}.$

From the Equation (21) we have $\eta^{*}=\frac{-b\pm \sqrt{b^{2}-4ac}}{2a}$.

Clearly, a is greater than zero. When $R_{0}>1,$ we have the following:

$$\begin{array}{cc} c & <0,\\ & ⟹\frac{-b+\sqrt{b^{2}-4ac}}{2a}>0\;\mathrm{and}\;\frac{-b-\sqrt{b^{2}-4ac}}{2a}<0. \end{array}$$

This shows that there is a unique endemic equilibrium point.

When $R_{0}<1,$ we have

$$\begin{array}{cc} & c>0,\\ & ⟹ac>0\;\mathrm{therefore}\;-4ac<0,\\ & ⟹\;\mathrm{if}\;b^{2}>4ac\;\mathrm{then}\;b^{2}-4ac>0,\\ & ⟹-b\pm \sqrt{b^{2}-4ac}<0,\\ & \;\mathrm{otherwise}\;\mathrm{no}\;\mathrm{real}\;\mathrm{roots}\;\mathrm{of}\;(21). \end{array}$$

#### Global Stability Analysis of the Endemic Equilibrium

Following [29,30,31], Theorem 6 below is established

**Theorem** **5.** *The unique endemic equilibrium of (3) is GAS in D if $R_{0}>1$, provided that*

(22) $$\left(1-\frac{\eta }{\eta^{*}}\right)\left(1-\frac{I\eta^{*}}{I^{*}\eta }\right)\geq 0$$

*and*

(23) $$\left(1-\frac{\eta }{\eta^{*}}\right)\left(1-\frac{H\eta^{*}}{H^{*}\eta }\right)\geq 0$$

*are satisfied.*

**Proof.**  In this case, we use the approach in [29,30,31] to establish the proof. If we consider a Lyapunov function:

(24) $$\begin{array}{c} L_{1}\left(t\right)=\omega_{1}\left(S_{1}-S_{1}^{*}-S_{1}^{*}\ln\frac{S_{1}}{S_{1}^{*}}\right)+\omega_{2}\left(S_{2}-S_{2}^{*}-S_{2}^{*}\ln\frac{S_{2}}{S_{2}^{*}}\right)+\omega_{3}\left(E-E^{*}-E^{*}\ln\frac{E}{E^{*}}\right)\\ +\omega_{4}\left(I-I^{*}-I^{*}\ln\frac{I}{I^{*}}\right)+\omega_{5}\left(H-H^{*}-H^{*}\ln\frac{H}{H^{*}}\right). \end{array}$$

where $\omega_{i}>0\left(i=1,2,3,4,5\right)$ are constants to be determined. It is easy to see that $L_{1}\geq 0$$\;\mathrm{for}\;\mathrm{all}\;S_{1},S_{2},E,I,H>0,\;\mathrm{and}\;L_{1}=0\Leftrightarrow \left(S_{1},S_{2},E,I,H\right)=\left(S_{1}^{*},S_{2}^{*},E^{*},I^{*},H^{*}\right)$. From (3), we have the solution below:

(25) $$\begin{array}{cc} \left(1-\rho \right)\Lambda -\left(\left(1-\sigma_{1}\right)\eta +\mu \right)S_{1} & =0,\\ \rho \Lambda -\left(\left(1-\sigma_{2}\right)\eta +\mu \right)S_{2} & =0,\\ \eta \left(\left(1-\sigma_{1}\right)S_{1}+\left(1-\sigma_{2}\right)S_{2}\right)-\left(\alpha +\mu \right)E & =0,\\ \alpha E-(\nu_{1}+\nu_{2}+\mu +\delta )I & =0,\\ \nu_{2}I-\left(\tau +\mu +\delta \right)H & =0,\\ \nu_{1}I+\tau H-\mu R & =0. \end{array}$$

We can differentiate $L_{1}$ along these solutions which is given by

(26) $$\begin{array}{c} \dot{L_{1}}\left(t\right)=\omega_{1}\left(1-\frac{S_{1}^{*}}{S_{1}}\right)\dot{S_{1}}+\omega_{2}\left(1-\frac{S_{2}^{*}}{S_{2}}\right)\dot{S_{2}}+\omega_{3}\left(1-\frac{E^{*}}{E}\right)\dot{E}\\ +\omega_{4}\left(1-\frac{I^{*}}{I}\right)\dot{I}+\omega_{5}\left(1-\frac{H^{*}}{H}\right)\dot{H}. \end{array}$$

By direct computation from (26), we have

(27) $$\begin{array}{cc} \omega_{1}\left(1-\frac{S_{1}^{*}}{S_{1}}\right)\dot{S_{1}} & =\omega_{1}\left(1-\frac{S_{1}^{*}}{S_{1}}\right)\left(\left(1-\rho \right)\Lambda -\left(\left(1-\sigma_{1}\right)\eta +\mu \right)S_{1}\right)\\ & =\omega_{1}\left(1-\frac{S_{1}^{*}}{S_{1}}\right)\left(\left(1-\rho \right)\Lambda -\left(1-\sigma_{1}\right)\eta S_{1}-\mu S_{1}\right)\\ & =\omega_{1}\left(1-\frac{S_{1}^{*}}{S_{1}}\right)\left(\left(1-\sigma_{1}\right)\eta^{*}S_{1}^{*}+\mu S_{1}^{*}-\left(1-\sigma_{1}\right)\eta S_{1}-\mu S_{1}\right)\\ & =\omega_{1}\left(1-\frac{S_{1}^{*}}{S_{1}}\right)\left(\left(1-\sigma_{1}\right)\eta^{*}S_{1}^{*}-\left(1-\sigma_{1}\right)\eta S_{1}-\mu S_{1}+\mu S_{1}^{*}\right)\\ & =\omega_{1}\left(1-\frac{S_{1}^{*}}{S_{1}}\right)\left(\left(1-\sigma_{1}\right)\eta^{*}S_{1}^{*}\left(1-\frac{\eta S_{1}}{\eta^{*}S_{1}^{*}}\right)-\mu \left(S_{1}-S_{1}^{*}\right)\right)\\ & =\omega_{1}\left(1-\sigma_{1}\right)\eta^{*}S_{1}^{*}\left(1-\frac{S_{1}^{*}}{S_{1}}\right)\left(1-\frac{\eta S_{1}}{\eta^{*}S_{1}^{*}}\right)-\omega_{1}\left(1-\frac{S_{1}^{*}}{S_{1}}\right)\mu \left(S_{1}-S_{1}^{*}\right)\\ & =\omega_{1}\left(1-\sigma_{1}\right)\eta^{*}S_{1}^{*}\left(1-\frac{S_{1}^{*}}{S_{1}}\right)\left(1-\frac{\eta S_{1}}{\eta^{*}S_{1}^{*}}\right)-\omega_{1}\mu \frac{{\left(S_{1}-S_{1}^{*}\right)}^{2}}{S_{1}}\\ & \leq \omega_{1}\left(1-\sigma_{1}\right)\eta^{*}S_{1}^{*}\left(1-\frac{S_{1}^{*}}{S_{1}}\right)\left(1-\frac{\eta S_{1}}{\eta^{*}S_{1}^{*}}\right)\\ & =\omega_{1}\left(1-\sigma_{1}\right)\eta^{*}S_{1}^{*}\left(1-\frac{\eta S_{1}}{\eta^{*}S_{1}^{*}}-\frac{S_{1}^{*}}{S_{1}}+\frac{\eta }{\eta^{*}}\right), \end{array}$$

and

(28) $$\begin{array}{cc} \omega_{2}\left(1-\frac{S_{2}^{*}}{S_{2}}\right)\dot{S_{2}} & =\omega_{2}\left(1-\frac{S_{2}^{*}}{S_{2}}\right)\left(\rho \Lambda -\left(\left(1-\sigma_{2}\right)\eta +\mu \right)S_{2}\right)\\ & =\omega_{2}\left(1-\frac{S_{2}^{*}}{S_{2}}\right)\left(\rho \Lambda -\left(1-\sigma_{2}\right)\eta S_{2}-\mu S_{2}\right)\\ & =\omega_{2}\left(1-\frac{S_{2}^{*}}{S_{2}}\right)\left(\left(1-\sigma_{2}\right)\eta^{*}S_{2}^{*}+\mu S_{2}^{*}-\left(1-\sigma_{2}\right)\eta S_{2}-\mu S_{2}\right)\\ & =\omega_{2}\left(1-\frac{S_{2}^{*}}{S_{2}}\right)\left(\left(1-\sigma_{2}\right)\eta^{*}S_{2}^{*}-\left(1-\sigma_{2}\right)\eta S_{2}-\mu S_{2}+\mu S_{2}^{*}\right)\\ & =\omega_{2}\left(1-\frac{S_{2}^{*}}{S_{2}}\right)\left(\left(1-\sigma_{2}\right)\eta^{*}S_{2}^{*}\left(1-\frac{\eta S_{2}}{\eta^{*}S_{2}^{*}}\right)-\mu \left(S_{1}-S_{2}^{*}\right)\right)\\ & =\omega_{2}\left(1-\sigma_{2}\right)\eta^{*}S_{2}^{*}\left(1-\frac{S_{2}^{*}}{S_{2}}\right)\left(1-\frac{\eta S_{2}}{\eta^{*}S_{2}^{*}}\right)-\omega_{2}\left(1-\frac{S_{2}^{*}}{S_{2}}\right)\mu \left(S_{2}-S_{2}^{*}\right)\\ & =\omega_{2}\left(1-\sigma_{2}\right)\eta^{*}S_{2}^{*}\left(1-\frac{S_{2}^{*}}{S_{2}}\right)\left(1-\frac{\eta S_{2}}{\eta^{*}S_{2}^{*}}\right)-\omega_{2}\mu \frac{{\left(S_{2}-S_{2}^{*}\right)}^{2}}{S_{2}}\\ & \leq \omega_{2}\left(1-\sigma_{2}\right)\eta^{*}S_{2}^{*}\left(1-\frac{S_{2}^{*}}{S_{2}}\right)\left(1-\frac{\eta S_{2}}{\eta^{*}S_{2}^{*}}\right)\\ & =\omega_{2}\left(1-\sigma_{2}\right)\eta^{*}S_{2}^{*}\left(1-\frac{\eta S_{2}}{\eta^{*}S_{2}^{*}}-\frac{S_{2}^{*}}{S_{2}}+\frac{\eta }{\eta^{*}}\right), \end{array}$$

and

(29) $$\begin{array}{cc} \omega_{3}\left(1-\frac{E^{*}}{E}\right)\dot{E} & =\omega_{3}\left(1-\frac{E^{*}}{E}\right)\left(\eta \left(1-\sigma_{1}\right)S_{1}+\eta \left(1-\sigma_{2}\right)S_{2}-k_{1}E\right)\\ & =\omega_{3}\left(1-\frac{E^{*}}{E}\right)(\eta \left(1-\sigma_{1}\right)S_{1}+\eta \left(1-\sigma_{2}\right)S_{2}\\ & -\left(\eta^{*}\left(1-\sigma_{1}\right)S_{1}^{*}\frac{E}{E^{*}}+\eta^{*}\left(1-\sigma_{2}\right)S_{2}^{*}\frac{E}{E^{*}}\right))\\ & =\omega_{3}\left(1-\frac{E^{*}}{E}\right)(\eta^{*}\left(1-\sigma_{1}\right)S_{1}^{*}\left(\frac{\eta S_{1}}{\eta^{*}S_{1}^{*}}-\frac{E}{E^{*}}\right)\\ & +\eta^{*}\left(1-\sigma_{2}\right)S_{2}^{*}\left(\frac{\eta S_{2}}{\eta^{*}S_{2}^{*}}-\frac{E}{E^{*}}\right))\\ & =\omega_{3}\eta^{*}\left(1-\sigma_{1}\right)S_{1}^{*}\left(1-\frac{E^{*}}{E}\right)\left(\frac{\eta S_{1}}{\eta^{*}S_{1}^{*}}-\frac{E}{E^{*}}\right)\\ & +\omega_{3}\eta^{*}\left(1-\sigma_{2}\right)S_{2}^{*}\left(1-\frac{E^{*}}{E}\right)\left(\frac{\eta S_{2}}{\eta^{*}S_{2}^{*}}-\frac{E}{E^{*}}\right)\\ & =\omega_{3}\left(1-\sigma_{1}\right)\eta^{*}S_{1}^{*}\left(\frac{\eta S_{1}}{\eta^{*}S_{1}^{*}}-\frac{E}{E^{*}}-\frac{\eta S_{1}E^{*}}{\eta^{*}S_{1}^{*}E}+1\right)\\ & +\omega_{3}\left(1-\sigma_{2}\right)\eta^{*}S_{2}^{*}\left(\frac{\eta S_{2}}{\eta^{*}S_{2}^{*}}-\frac{E}{E^{*}}-\frac{\eta S_{2}E^{*}}{\eta^{*}S_{2}^{*}E}+1\right), \end{array}$$

and

(30) $$\begin{array}{cc} \omega_{4}\left(1-\frac{I^{*}}{I}\right)\dot{I} & =\omega_{4}\left(1-\frac{I^{*}}{I}\right)\left(\alpha E-k_{3}I\right)\\ & =\omega_{4}\left(1-\frac{I^{*}}{I}\right)\left(\alpha E-\alpha E^{*}\frac{I}{I^{*}}\right)\\ & =\omega_{4}\alpha E^{*}\left(1-\frac{I^{*}}{I}\right)\left(\frac{E}{E^{*}}-\frac{I}{I^{*}}\right)\\ & =\omega_{4}\alpha E^{*}\left(\frac{E}{E^{*}}-\frac{I}{I^{*}}-\frac{I^{*}E}{IE^{*}}+1\right), \end{array}$$

and

(31) $$\begin{array}{cc} \omega_{5}\left(1-\frac{H^{*}}{H}\right)\dot{H} & =\omega_{5}\left(1-\frac{H^{*}}{H}\right)\left(\nu_{2}I-k_{4}H\right)\\ & =\omega_{5}\left(1-\frac{H^{*}}{H}\right)\left(\nu_{2}I-\nu_{2}I^{*}\frac{H}{H^{*}}\right)\\ & =\omega_{5}\nu_{2}I^{*}\left(1-\frac{H^{*}}{H}\right)\left(\frac{I}{I^{*}}-\frac{H}{H^{*}}\right)\\ & =\omega_{5}\nu_{2}I^{*}\left(\frac{I}{I^{*}}-\frac{H}{H^{*}}-\frac{H^{*}I}{HI^{*}}+1\right). \end{array}$$

Substituting $\omega_{1}=\omega_{2}=\omega_{3}=1,\omega_{4}=\frac{\eta^{*}\left(\left(1-\sigma_{1}\right)S_{1}^{*}+\left(1-\sigma_{2}\right)S_{2}^{*}\right)}{\alpha E^{*}},\;\mathrm{and}\;\omega_{5}=\frac{\eta^{*}\left(1-\sigma_{2}\right)S_{2}^{*}}{\nu_{2}I^{*}}$, and (27)–(31) into (26), we have

(32) $$\begin{array}{c} \dot{L_{1}}\left(t\right)\leq \left(1-\sigma_{1}\right)\eta^{*}S_{1}^{*}\left(2-\frac{S_{1}^{*}}{S_{1}}-\frac{E}{E^{*}}-\frac{\eta S_{1}E^{*}}{\eta^{*}S_{1}^{*}E}+\frac{\eta }{\eta^{*}}\right)\\ +\left(1-\sigma_{2}\right)\eta^{*}S_{2}^{*}\left(2-\frac{S_{2}^{*}}{S_{2}}-\frac{E}{E^{*}}-\frac{\eta S_{2}E^{*}}{\eta^{*}S_{2}^{*}E}+\frac{\eta }{\eta^{*}}\right)\\ +\left(1-\sigma_{1}\right)\eta^{*}S_{1}^{*}\left(\frac{E}{E^{*}}-\frac{I}{I^{*}}-\frac{I^{*}E}{IE^{*}}+1\right)\\ +\left(1-\sigma_{2}\right)\eta^{*}S_{2}^{*}\left(\frac{E}{E^{*}}-\frac{I}{I^{*}}-\frac{I^{*}E}{IE^{*}}+1\right)\\ +\left(1-\sigma_{2}\right)\eta^{*}S_{2}^{*}\left(\frac{I}{I^{*}}-\frac{H}{H^{*}}-\frac{H^{*}I}{HI^{*}}+1\right). \end{array}$$

Following the idea in [31,32], suppose we have a function defined as χ(x)=1−x+ln(x), if x>0, we have χ(x)≤0, and if x=1, we have χ(1)=0.Thus,x−1≥ln(x)forx>0 Using this relation we find that

(33) $$\begin{array}{c} \left(2-\frac{S_{1}^{*}}{S_{1}}-\frac{E}{E^{*}}-\frac{\eta S_{1}E^{*}}{\eta^{*}S_{1}^{*}E}+\frac{\eta }{\eta^{*}}\right)=\\ \left(-1+\frac{\eta }{\eta^{*}}\right)\left(1-\frac{I\eta^{*}}{I^{*}\eta }\right)+3-\frac{S_{1}^{*}}{S_{1}}-\frac{\eta S_{1}E^{*}}{\eta^{*}S_{1}^{*}E}-\frac{I\eta^{*}}{I^{*}\eta }-\frac{E}{E^{*}}+\frac{I}{I^{*}}\\ \leq -\left(\frac{S_{1}^{*}}{S_{1}}-1\right)-\left(\frac{\eta S_{1}E^{*}}{\eta^{*}S_{1}^{*}E}-1\right)-\left(\frac{I\eta^{*}}{I^{*}\eta }-1\right)-\frac{E}{E^{*}}+\frac{I}{I^{*}}\\ \leq -\ln\left(\frac{S_{1}^{*}\eta S_{1}E^{*}I\eta^{*}}{S_{1}\eta^{*}S_{1}^{*}EI^{*}\eta }\right)-\frac{E}{E^{*}}+\frac{I}{I^{*}}\\ =\frac{I}{I^{*}}-\ln\left(\frac{I}{I^{*}}\right)+\ln\left(\frac{E}{E^{*}}\right)-\frac{E}{E^{*}}. \end{array}$$

Likewise,

(34) $$\begin{array}{c} \left(2-\frac{S_{2}^{*}}{S_{2}}-\frac{E}{E^{*}}-\frac{\eta S_{2}E^{*}}{\eta^{*}S_{2}^{*}E}+\frac{\eta }{\eta^{*}}\right)=\\ \left(-1+\frac{\eta }{\eta^{*}}\right)\left(1-\frac{H\eta^{*}}{H^{*}\eta }\right)+3-\frac{S_{2}^{*}}{S_{2}}-\frac{\eta S_{2}E^{*}}{\eta^{*}S_{2}^{*}E}-\frac{H\eta^{*}}{H^{*}\eta }-\frac{E}{E^{*}}+\frac{H}{H^{*}}\\ \leq -\left(\frac{S_{2}^{*}}{S_{2}}-1\right)-\left(\frac{\eta S_{2}E^{*}}{\eta^{*}S_{2}^{*}E}-1\right)-\left(\frac{H\eta^{*}}{H^{*}\eta }-1\right)-\frac{E}{E^{*}}+\frac{H}{H^{*}}\\ \leq -\ln\left(\frac{S_{2}^{*}\eta S_{2}E^{*}H\eta^{*}}{S_{2}\eta^{*}S_{2}^{*}EH^{*}\eta }\right)-\frac{E}{E^{*}}+\frac{H}{H^{*}}\\ =\frac{H}{H^{*}}-\ln\left(\frac{H}{H^{*}}\right)+\ln\left(\frac{E}{E^{*}}\right)-\frac{E}{E^{*}}. \end{array}$$

Meanwhile, one can still verify that

(35) $$\begin{array}{c} \left(\frac{E}{E^{*}}-\frac{I}{I^{*}}-\frac{I^{*}E}{IE^{*}}+1\right)=-\left(\frac{I^{*}E}{IE*}-1\right)+\frac{E}{E*}-\frac{I}{I*}\leq -\ln\left(\frac{I^{*}E}{IE*}\right)+\frac{E}{E*}-\frac{I}{I*}\\ \leq -\ln\left(\frac{I^{*}}{I}\right)-\ln\left(\frac{E}{E*}\right)+\frac{E}{E*}-\frac{I}{I*}=\frac{E}{E*}-\ln\left(\frac{E}{E*}\right)-\frac{I}{I*}+\ln\left(\frac{I}{I*}\right). \end{array}$$

Similarly, we have

(36) $$\begin{array}{c} \left(\frac{I}{I^{*}}-\frac{H}{H^{*}}-\frac{H^{*}I}{HI^{*}}+1\right)=-\left(\frac{H^{*}I}{HI*}-1\right)+\frac{I}{I*}-\frac{H}{H*}\leq -\ln\left(\frac{H^{*}I}{HI*}\right)+\frac{I}{I*}-\frac{H}{H*}\\ \leq -\ln\left(\frac{H^{*}}{H}\right)-\ln\left(\frac{I}{I*}\right)+\frac{I}{I*}-\frac{H}{H*}=\frac{I}{I*}-\ln\left(\frac{I}{I*}\right)-\frac{H}{H*}+\ln\left(\frac{H}{H*}\right). \end{array}$$

Substituting (33)–(36) in (32) we have,

(37) $$\begin{array}{c} \dot{L_{1}}\left(t\right)\leq \left(1-\sigma_{1}\right)\eta^{*}S_{1}^{*}\left(\frac{I}{I^{*}}-\ln\left(\frac{I}{I^{*}}\right)+\ln\left(\frac{E}{E^{*}}\right)-\frac{E}{E^{*}}\right)+\\ \left(1-\sigma_{2}\right)\eta^{*}S_{2}^{*}\left(\frac{H}{H^{*}}-\ln\left(\frac{H}{H^{*}}\right)+\ln\left(\frac{E}{E^{*}}\right)-\frac{E}{E^{*}}\right)+\\ \left(1-\sigma_{1}\right)\eta^{*}S_{1}^{*}\left(\frac{E}{E*}-\ln\left(\frac{E}{E*}\right)-\frac{I}{I*}+\ln\left(\frac{I}{I*}\right)\right)+\\ \left(1-\sigma_{2}\right)\eta^{*}S_{2}^{*}\left(\frac{E}{E*}-\ln\left(\frac{E}{E*}\right)-\frac{I}{I*}+\ln\left(\frac{I}{I*}\right)\right)+\\ \left(1-\sigma_{2}\right)\eta^{*}S_{2}^{*}\left(\frac{I}{I*}-\ln\left(\frac{I}{I*}\right)-\frac{H}{H*}+\ln\left(\frac{H}{H*}\right)\right). \end{array}$$

Hence, (27)–(37) ensure that $\frac{dL_{1}}{dt}\leq 0$. It is easy to see that $\frac{dL_{1}}{dt}=0$ holds only for $S_{1}=S_{1}^{*},S_{2}=S_{2}^{*},E=E^{*},I=I^{*},H=H^{*},\;\mathrm{and}\;R=R^{*}$. In a similar manner in [28], every solutions of our model system (3) with initial conditions in D approaches the stable EE as t⟶∞. Hence, EE is GAS equilibrium of (3) on D. □

## 4. Numerical Simulations

In addition to the theoretical results from the model analysis, it is also crucial that the model equations are simulated in order to gain an understanding of the model. This simulations were conducted using MATLAB R2022b on a mid-range personal laptop that features an i7 processor and 8GB of RAM on a Windows 11 operating system. In the Supplementary Materials, a copy of the simulation code that was used can be found. While it is a challenge to find suitable data for model simulation, the lack of an appropriate answer to this question continues to be a major problem. We used parameters provided in the existing literature to simulate and perform a sensitivity analysis of our model. Occasionally, we assumed the values which can be seen in Table 3.

**Figure 2:**
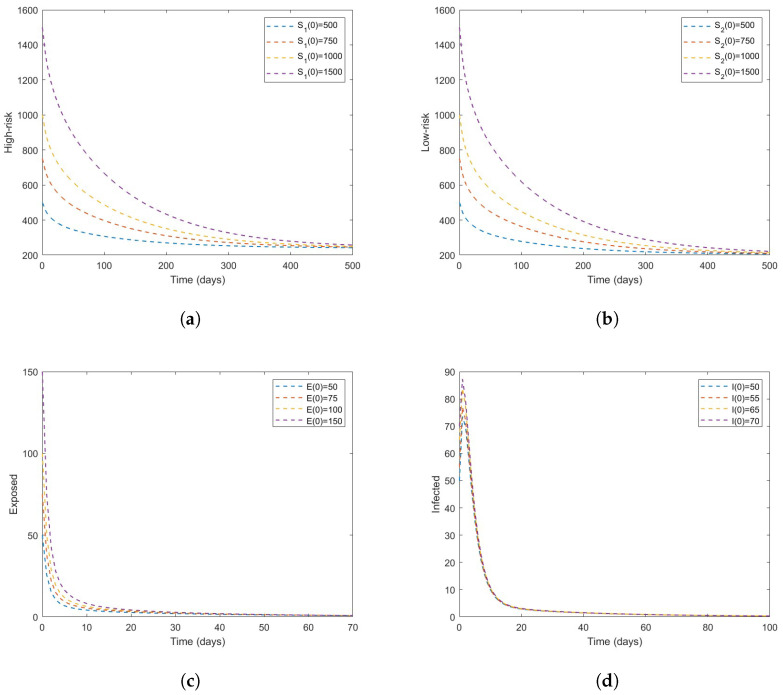
Time series plot of the model (3) with different initial conditions (described by a color scheme). The parameter values are given in Table 3 with $\beta =2693\;\mathrm{so}\;\mathrm{that}\;R_{0}=0.9380<1$; (**a**) the high-risk susceptible, (**b**) the low-risk susceptible, (**c**) exposed, and (**d**) infectious.

**Figure 3:**
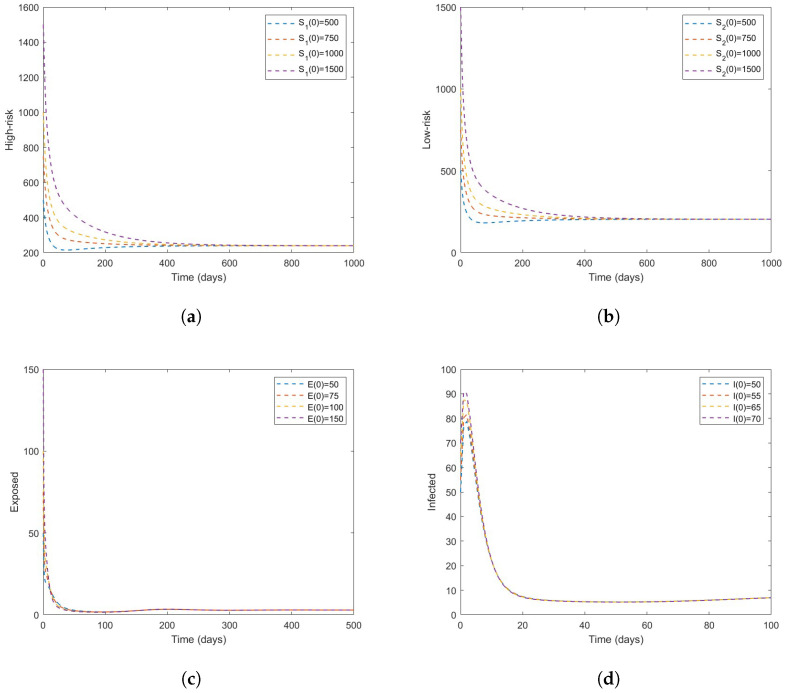
Time series plot of the model (3) with different initial conditions (described by a color scheme). The parameter values are given in Table 3 with $\beta =0.6886\;\mathrm{so}\;\mathrm{that}\;R_{0}=2.3986>1$; (**a**) the high-risk susceptible, (**b**) the low-risk susceptible, (**c**) exposed, and (**d**) infectious.

We begin the numerical simulation of our model by first letting β=0.2693 so that $R_{0}=0.9380<1$ for various initial conditions. When $R_{0}<1$ Figure 2a–d shows that the system has a DFE that is asymptotically stable which supports the result stated in Theorem 3. In Figure 3, we examine the scenario in which $R_{0}=2.3986>1$ for various initial conditions. When $R_{0}>1$ Figure 3a–d shows that the system has a DFE and it is unstable whenever $R_{0}>1$.

### Sensitivity Analysis

In mathematical modeling, numerous parameter values are uncertain, this could be due to incorrect parameter estimation and uncertainty regarding the accurate values of the parameters. Therefore, it is reasonable to carry out sampling and sensitivity analysis to identify the parameters that significantly affect the output of our model. We used a Sampling and Sensitivity Analysis Tool (SaSAT) in [36] to recognise these parameters. This is an efficient tool that enables us to analyze the sensitivity. We first have values and boundaries assigned to our parameters as in Table 3. We then applied uniform probability distributions to each of the parameters, in accordance with the suggestion of [36]. We evaluated Partial Rank Correlation Coefficients (PRCC) with 1000 samples for each parameter per run, which was resulted from the Latin hypercube sampling (LHS).

**Figure 4:**
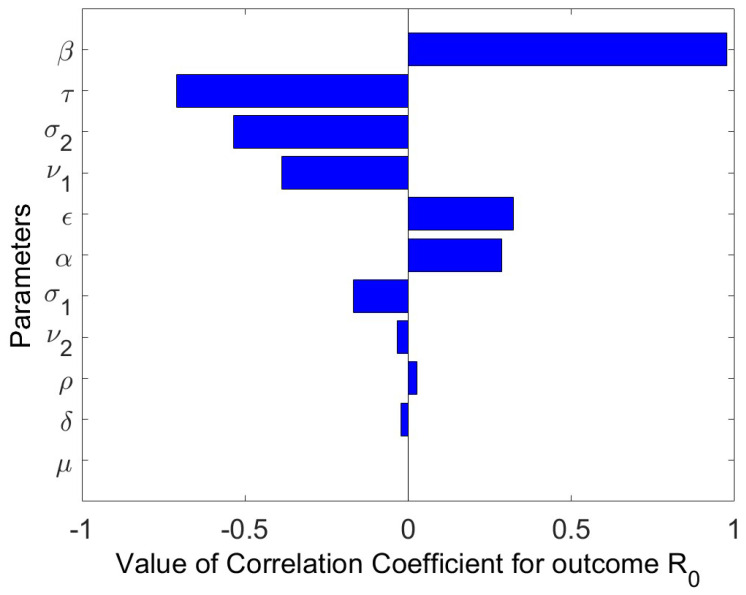
Tornado plot for the model parameter sensitivities affecting the basic reproduction number $R_{0}$.

**Figure 5:**
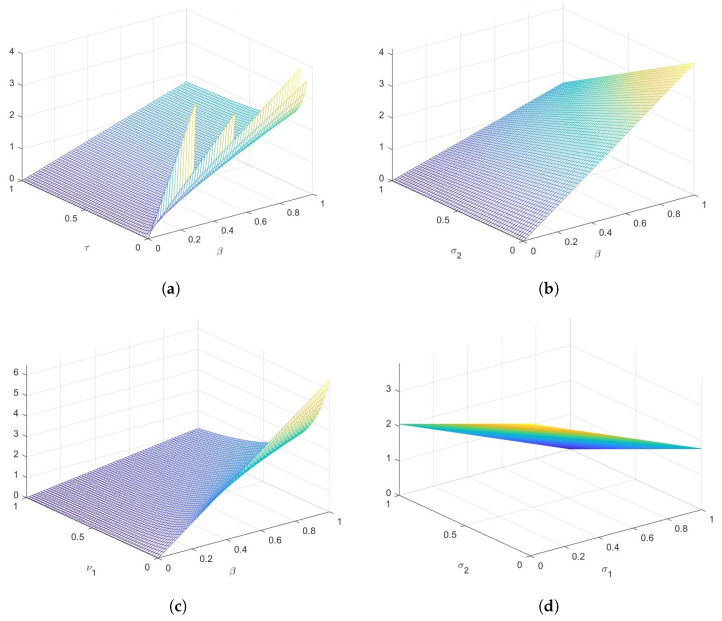

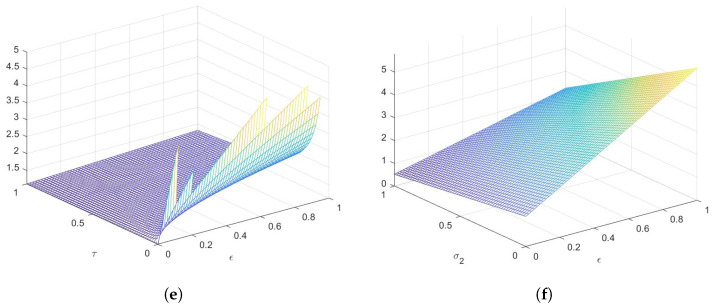
Response surface plot showing the change in behavior of $R_{0}$ while varying the value of the most sensitive parameters. (**a**) Plot of $R_{0}\;\mathrm{vs}.\;\tau \;\mathrm{and}\;\beta$. (**b**) Plot of $R_{0}\;\mathrm{vs}.\;\tau \;\mathrm{and}\;\mu$. (**c**) Plot of $R_{0}\;\mathrm{vs}.\;\nu_{1}\;\mathrm{and}\;\mu$. (**d**) Plot of $R_{0}\;\mathrm{vs}.\;\sigma_{2}\;\mathrm{and}\;\sigma_{1}$. (**e**) Plot of $R_{0}\;\mathrm{vs}.\;\epsilon \;\mathrm{and}\;\beta$ (**f**) Plot of $R_{0}\;\mathrm{vs}.\;\nu_{2}\;\mathrm{and}\;\beta$.

Figure 4 shows how varying the parameters of model (3) changes the behavior of $R_{0}$. The five (5) most sensitive parameters affecting $R_{0}$ are $\beta ,\tau ,\sigma_{2},\nu_{1},\;\mathrm{and}\;\epsilon$. Rising the value of the recovery rate of the hospitalized individuals, rate of reduction in infectiousness of the low-risk susceptible which is proportional to rising the value of $\sigma_{1}$ might cause the value of $R_{0}$ to drop. Meanwhile, reducing the contact rate, hospital inefficacy might also cause the value of $R_{0}$ to drop significantly. This study demonstrate that isolating and hospitalizing the infected persons reduces the spread of infection. Figure 5 shows the change in behavior of $R_{0}$ while varying the value of the most sensitive parameters.

## 5. Extended Model with Vaccination

Vaccine effectiveness has so far been the driving force in the dynamics of COVID-19 transmission. In this section, we carried out numerical simulations to test for various situations in relation to the parameters under close examination. We propose two main parameters ω, which indicates the level of vaccination for the high-risk population, and $\nu_{3}$, which represents the effectiveness of the vaccine among the vaccinated individuals in the low-risk population.

In summary, as a result of incorporating an imperfect COVID-19 vaccine we believe:

**i.**  The number of high-risk individuals reduces.
**ii.**  Infections can be prevented with some degree of efficacy.

Next, we see the effect of vaccination and its effectiveness on the dynamics of COVID-19 by simulating the related vaccine parameters of the model (3) over time.

### The Effect of Vaccine on the Disease Dynamics

Some high-risk individuals after being vaccinated at the rate ω move to the low-risk class and further move to the recovered class depending on the vaccine efficacy, $\nu_{3}=0.01,\nu_{3}=0.05,\nu_{3}=0.1,\nu_{3}=0.15,\nu_{3}=0.2,\;\mathrm{and}\;\nu_{3}=0.3$.

**Figure 6:**
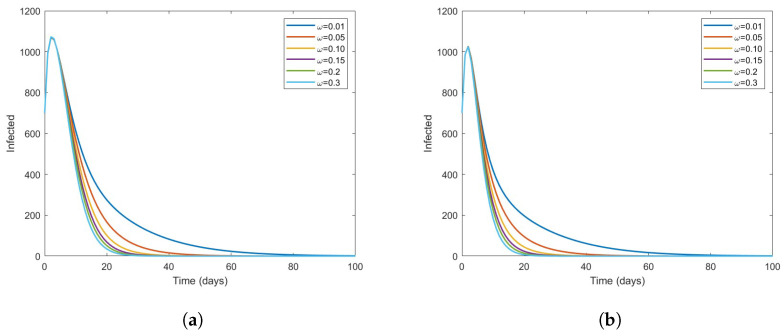
The effect of the vaccine on infected COVID-19 individuals for $\nu_{3}=0.25\;\mathrm{and}\;\nu_{3}=0.75$. (**a**) $\nu_{3}=0.25$. (**b**) $\nu_{3}=0.75$.

**Figure 7:**
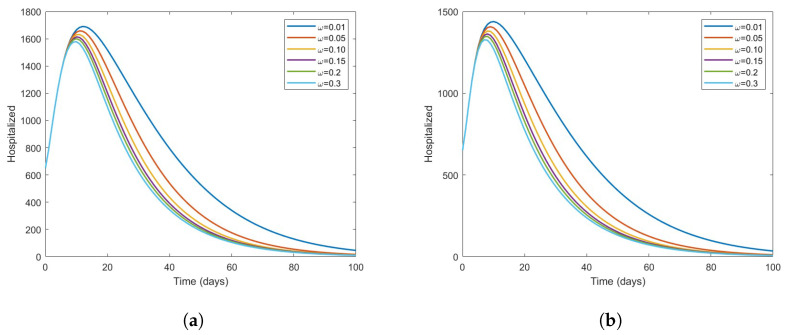
The effect of the vaccine on hospitalized COVID-19 individuals for $\nu_{3}=0.25\;\mathrm{and}\;\nu_{3}=0.75$. (**a**) $\nu_{3}=0.25$. (**b**) $\nu_{3}=0.75$.

In Figure 6, we can see that as vaccination coverage increases, there is a striking decline in the number of infected individuals at each level of effectiveness of the vaccine. In Figure 7, we can see that the hospitalized population decreases relatively quickly over time at various levels of $\nu_{3}$. Therefore, improving the vaccination rates for people at high risk will result in a decrease in the transmission of COVID-19 in the future.

**Figure 8:**
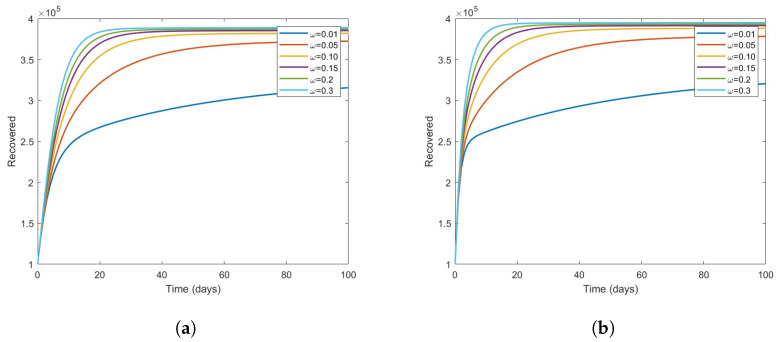
The effect of the vaccine on recovered individuals for $\nu_{3}=0.25\;\mathrm{and}\;\nu_{3}=0.75$. (**a**) $\nu_{3}=0.25$. (**b**) $\nu_{3}=0.75$.

A look at Figure 8 illustrates, on the other hand, what the effects of vaccination may be on the recovered class, it can be concluded that the population of the recovered class increases as the value of ω increases from 0.01to0.3.

## 6. Discussion and Conclusions

As it stands now, there is still no specific drug for COVID-19, so many treatment guidelines were developed in the literature based on patient treatment records for physicians and healthcare personnel as a guide for decision-making in the treatment of patients with COVID-19. Understanding the influence of the NPIs on both low and high-risk individuals, the impact of the vaccine on the high-risk population can help us inform public health policy as it was discussed in [37]. It is equally vital to evaluate the impact of hospital efficacy/inefficacy.

In this paper, we present a model which explains the transmission and spread of COVID-19, incorporating a fraction ρ low-risk and the remaining (1−ρ) high-risk of the human population. The model also captures NPIs by both low- and high-risk individuals, to reduce infection transmission and insisted that the high-risk individuals take more intervention than the low-risk, similar to what is mentioned in [38]. The model was later extended by incorporating the vaccination of some high-risk individuals and vaccine efficacy. This study has been able to draw some important conclusions both mathematically and biologically, which are summarized below.

Many models of the SEIR form in literature, see [21,22,39,40], did not take into consideration the segregation in the susceptible class into low- and high-risk individuals. However, considering this division is essential in providing more insight into the transmission dynamics of the disease, as well as guiding the relevant authorities in introducing and providing plans to curtail the spread of the disease.

**(i)**  A GAS DFE occurs in the model without vaccination every time the associated basic reproduction number is less than 1 and there are multiple endemic equilibria, which is a GAS in a particular case.
**(ii)**  Based on numerical simulations it is indicated that if some high-risk individuals were vaccinated and moved to low-risk, the disease could be reduced to a minimum. However, this is dependent on the coverage of vaccinations.
**(iii)**  The Latin Hypercube Sampling to create 1000 samples and the resulting Partial Rank Correlation Coefficients (PRCCs) were used to perform a sensitivity study of the model parameter values. A tornado plot is used to visually display the results. The parameters τ, (hospitalized recovery rate), $\sigma_{1,2}$, (reduction in infectiousness of risk individuals), according to sensitivity analysis, greatly lessen an epidemic if increased. However, if the rates of person-to-person interaction and hospital inefficacy are reduced, transmission also decreases.

In order to stop the epidemic, it is crucial to improve vaccination of the high-risk individuals. Law enforcement must also be strengthened in order to restrict undocumented immigration.

### Future Work Directions

- Some new models with a different approach of incidence fraction can be proposed.
- Several dynamical features of COVID-19 were captured by our model, though other population compartments might be added and furthermore implement optimal control strategies when having access to more detailed and authentic COVID-19 data.

## Figures and Tables

**Table 1:** Description of the variables.

Variables	Description
$S_{1}$	Number of high-risk susceptible population
$S_{2}$	Number of low-risk susceptible population
*E*	Number of exposed population
*I*	Number of infected population
*H*	Number of hospitalize population
*R*	Number of recovered population
*N*	Total human population

**Table 2:** Description of the parameters.

Parameter	Description
Λ	Recruitment rate
β	Effective contact rate
ρ	Fraction of newly recruited individuals moving to $S_{2}$
$\sigma_{1}$	Rate of reduction in infectiousness in $S_{1}$
$\sigma_{2}$	Rate of reduction in infectiousness in $S_{2}$
μ	The natural death rate
α	The rate of progression from exposed population to infected population
ϵ	The rate of hospital inefficacy
$\nu_{1}$	The recovery rate of infected in
$\nu_{2}$	The hospitalization rate of infected individuals
τ	The recovery rate of the hospitalized individuals
δ	The COVID-19 induced mortality rate

**Table 3:** Parameter, values, and boundary.

Parameters	Values/Source	Boundary/Source
β	0.6886, assumed	[0,1], estimated
ρ	0.7, assumed	[0.5,1], estimated
$\sigma_{1}$	0.4521, [33]	[0.213,0.51], estimated
$\sigma_{2}$	0.2757, [33]	[0.2,0.5], estimated
μ	0.0079, assumed	[0.0074,0.008], estimated
α	0.1667, [19]	[0,1], estimated
ϵ	0.0075, assumed	[0.3,0.4], estimated
$\nu_{1}$	0.1, [34]	[0.1,0.2], estimated
$\nu_{2}$	0.2, [34]	[0.195,0.39], [35]
τ	0.005, assumed	[0.03,0.08], estimated
δ	0.0015 assumed	[0.0013,0.0023], estimated

## Data Availability

There are no underlying data.

## Supplementary Materials

The following supporting information can be downloaded at: https://www.mdpi.com/article/10.3390/vaccines11010003/s1.

## Abbreviations

The following abbreviations are used in this manuscript:

COVID-19	Coronavirus disease 2019
NPIs	Non-pharmaceutical interventions against COVID-19
DFE	Disease-free equilibrium
EE	Endemic equilibrium
LAS	Locally asymptotically stable
GAS	Globally asymptotically stable
SaSAT	Sampling and sensitivity analysis tool
PRCC	Partial rank correlation coefficient
LHS	Latin hypercube sampling
ASQ	Alternative state quarantine
