# Supplementary material for: COVID-19 Model with High- and Low-Risk Susceptible Population Incorporating the Effect of Vaccines
Source: Vaccines (Basel). 2022 Dec 20;11(1):3. doi: 10.3390/vaccines11010003 (PMC9861103; doi:10.3390/vaccines11010003)
Supplement: Supplementary file 1 [file vaccines-11-00003-s001.zip › vaccines-2080022-supplementary.pdf]

MATLAB Simulation Code

CODE 1

```
% Diffential system

function dydt=elhas(t,y,varphi)
Lambda=3.5;
betaa=0.6886;
rhoo=0.46;
sigma_1=0.4521;
sigma_2=0.2757;
muu=0.0079;
alphaa=0.767;
epsilonn=0.35;
nu_1=0.18;
nu_2=0.2;
tauu=0.05;
deltaa=0.0015;
%N=480000; %high-risk and low risk
%N=380; %exposed
N=200; %infected


dydt=zeros(6,1); % zeros(number of ODE, 1)
dydt(1) = (1-rhoo)*Lambda-(1-sigma_1)*(betaa*(y(4)+epsilonn*y(5))/N)*y(1)-
muu*y(1);
dydt(2) = rhoo*Lambda-(1-sigma_2)*(betaa*(y(4)+epsilonn*y(5))/N)*y(2)-muu*y(2);
dydt(3) = (1-sigma_1)*betaa*(y(4)+epsilonn*y(5))*y(1)/N+(1-
sigma_2)*betaa*(y(4)+epsilonn*y(5))*y(2)/N-(alphaa+muu)*y(3);
dydt(4) = alphaa*y(3)-(nu_1+nu_2+muu+deltaa)*y(4);
dydt(5) = nu_2*y(4)-(tauu+muu+deltaa)*y(5);
dydt(6) = nu_1*y(4)+tauu*y(5)-muu*y(5);
```

CODE 1 Driver

```
%main file for dengue function (paper for Mathematical population studies with
colors)


  for i=1:4

%    y1=[500 750 1000 1500];
%    y0=[y1(i), 150000, 100000, 800, 750, 1000];

%     y2=[500 750 1000 1500];
%     y0=[150000, y2(i), 100000, 800, 750, 1000];

%     y3=[50 75 100 150];
%     y0=[150, 150, y3(i), 10, 10, 10];

   y4=[50 55 65 70];
   y0=[50, 50, 90, y4(i), 20, 20];

 %tspan=[0:1000]; %high-risk and low-risk
```

```
 %tspan=[0:500]; % exposed
 tspan=[0:100];
 [t,y] = ode45(@elhas2,tspan,y0,[],y4(i));

 figure(1)
 plot(t,y(:,4),'--','LineWidth',1);hold on;
 xlabel('Time (days)');
 ylabel('Infected');

 end
 hold off;
```

CODE 2

```
% Diffential system

function dydt=elhas(t,y,omegaa)
% Parameters
Lambda=3.5;
betaa=0.6886;
rhoo=0.46;
sigma_1=0.4521;
sigma_2=0.2757;
muu=0.0079;
alphaa=0.767;
epsilonn=0.35;
%varphi=1;
%varphi=0.25;
varphi=0.75;
%N=0.21;
nu_1=0.18;
nu_2=0.2;
tauu=0.05;
deltaa=0.0015;
%N=480000;
N=402350;

dydt=zeros(6,1); % zeros(number of ODE, 1)
dydt(1) = (1-rhoo)*Lambda-(1-sigma_1)*(betaa*(y(4)+epsilonn*y(5))/N)*y(1)-
(muu+omegaa)*y(1);
dydt(2) = rhoo*Lambda+omegaa*y(1)-(1-sigma_2)*(betaa*(y(4)+epsilonn*y(5))/N)*y(2)-
(muu+varphi)*y(2);
dydt(3) = (1-sigma_1)*betaa*(y(4)+epsilonn*y(5))*y(1)/N+(1-
sigma_2)*betaa*(y(4)+epsilonn*y(5))*y(2)/N-(alphaa+muu)*y(3);
dydt(4) = alphaa*y(3)-(nu_1+nu_2+muu+deltaa)*y(4);
dydt(5) = nu_2*y(4)-(tauu+muu+deltaa)*y(5);
dydt(6) = nu_1*y(4)+tauu*y(5)-muu*y(5)+varphi*y(2);
```

CODE 2 Driver

```
%main file for dengue function (paper for Mathematical population studies with
colors)

y0=[150000, 150000, 1000, 700, 650, 100000];
tspan=[0:100];
omegaa =[0.01 0.05 0.1 0.15 0.2 0.3];

 for i=1:6
```

```
 tspan=[0:100];
 [t,y] = ode45(@elhas,tspan,y0,[],omegaa(i));

%  figure(1)
%  plot(t,y(:,1),'-','LineWidth',1.5);hold on;
%  xlabel('Time (days)');
%  ylabel('High-risk');
%
legend('omega=0.01','omega=0.04','omega=0.08','omega=0.12','omega=0.2','omega=0.3'
);
%
%  figure(2)
%  plot(t,y(:,2),'-','LineWidth',1.5);hold on;
%  xlabel('Time (days)');
%  ylabel('Low-risk');
%
legend('omega=0.01','omega=0.04','omega=0.08','omega=0.12','omega=0.2','omega=0.3'
);

%  figure(3)
%  plot(t,y(:,3),'-','LineWidth',1.5);hold on;
%  xlabel('Time (days)');
%  ylabel('Exposed');
%
legend('omega=0.01','omega=0.05','omega=0.10','omega=0.15','omega=0.2','omega=0.3'
);

%  figure(4)
%  plot(t,y(:,4),'-','LineWidth',1.5);hold on;
%  xlabel('Time (days)');
%  ylabel('Infected');
%
legend('omega=0.01','omega=0.05','omega=0.10','omega=0.15','omega=0.2','omega=0.3'
);

%  figure(5)
%  plot(t,y(:,5),'-','LineWidth',1.5);hold on;
%  xlabel('Time (days)');
%  ylabel('Hospitalized');
%
legend('omega=0.01','omega=0.05','omega=0.10','omega=0.15','omega=0.2','omega=0.3'
);

 figure(6)
 plot(t,y(:,6),'-','LineWidth',1.5);hold on;
 xlabel('Time (days)');
 ylabel('Recovered');

legend('omega=0.01','omega=0.05','omega=0.10','omega=0.15','omega=0.2','omega=0.3'
);
 end
 hold off;
```

Surface Plot Code

```
clear all
beta=0.02;
rho=0.7;
sigma_1=0.4521;
```

```
sigma_2=0.2757;
mu=0.0079;
alpha=0.1667;
epsilon=0.0075;
nu_1=0.1;
nu_2=0.2;
tau=0.005;
delta=0.0015;
syms tau beta
%syms mu tau
%syms mu nu_1
%syms sigma_1 sigma_2
%syms epsilon beta
%syms nu_2 beta
R0=beta*alpha*(epsilon*nu_2+delta+mu+tau)*((sigma_1-sigma_2)*rho+(1-
sigma_1))./((nu_1+nu_2+mu+delta).*(alpha+mu).*(tau+mu+delta))
ezmesh(R0,[0 1],[0 1])
zlabel('R_0')
```
